# Standardising image registration and dose mapping for thoracic reirradiation: A national multi-centre benchmarking study^☆^

**DOI:** 10.1016/j.phro.2026.100913

**Published:** 2026-01-29

**Authors:** Hella Sand, Dennis Arp, Maria F Jensen, Marianne M Knap, Christina Larsen, Mikkel D Lund, Morten Nielsen, Wiviann Ottosson, Ane Appelt, Lone Hoffmann

**Affiliations:** a Department of Oncology, Aalborg University Hospital, Aalborg, Denmark; b Danish Centre for Particle Therapy, Aarhus University Hospital, Aarhus, Denmark; c Department of Oncology, Aarhus University Hospital, Aarhus, Denmark; d Department of Oncology, Copenhagen University Hospital – Herlev and Gentofte, Herlev, Denmark; e Department of Oncology, Vejle Hospital, University Hospital of Southern Denmark, Vejle, Denmark; f Department of Oncology, Odense University Hospital, Odense, Denmark; g Leeds Institute of Medical Research, University of Leeds, Leeds, United Kingdom; h Department of Clinical Medicine, Faculty of Health Sciences, Aarhus University, Aarhus, Denmark

**Keywords:** Radiotherapy, Thoracic reirradiation, Multicentre study, Quality assurance, Deformable image registration (DIR), Rigid image registration (RIR), Image registration variability, Lung cancer, Dose mapping, DVH analysis

## Abstract

**Background and purpose:**

Safe delivery of high-dose reirradiation depends on robust evaluation of previously delivered dose on current patient anatomy. This study aims to assess the variability of 1) rigid (RIR) and deformable (DIR) image registration, and 2) dose-mapping based on RIR or DIR, as part of the pre-trial quality assurance for CURE Lung, a Scandinavian thoracic reirradiation trial.

**Materials and methods:**

Seven lung cancer cases, treated with curatively intended reirradiation near a previously irradiated region, were distributed to six Danish centres. Organs-at-risk (OARs) were delineated on CT images of previous (CT_prev_) and current treatment (CT_current_). RIR and DIR from CT_prev_ to CT_current_ were performed for all cases at each centre, and doses were mapped from CT_prev_ to CT_current_ using both methods. Mapped and current physical doses were converted to equieffective doses (EQD2), and cumulative EQD2-doses were generated on CT_current_. Inter-centre variation was assessed using volume coefficients and distance measurements. A linear mixed-effects model tested the effect of registration method on OAR alignment, near-maximum and volumetric EQD2-dose constraints from CURE Lung, and variability in DVH bands.

**Results:**

DIR significantly reduced the geometrical variations for all OARs, with mean surface distances <4.4 mm. Near-maximum EQD2-doses overall had less variability with DIR compared to RIR. No significant differences were found for the full DVH bands.

**Conclusion:**

DIR provides significantly better consistency in transfer of OAR from CT_prev_ to CT_current_ and reduces variability in EQD2-dose evaluations compared to RIR. This supports preferential use of DIR for reirradiation planning in lung cancer.

## Highlights

• Lung reirradiation dose accumulation across six centres.
• Image registration performed using three software solutions.
• DIR (deformable) improved organ-at-risk alignment compared to RIR (rigid) methods.
• Applying CURE Lung constraints DIR gave statistically less dose variation than RIR.
• Findings support standardised use of DIR for safe lung cancer reirradiation.

## Graphical abstract

**Figure:**
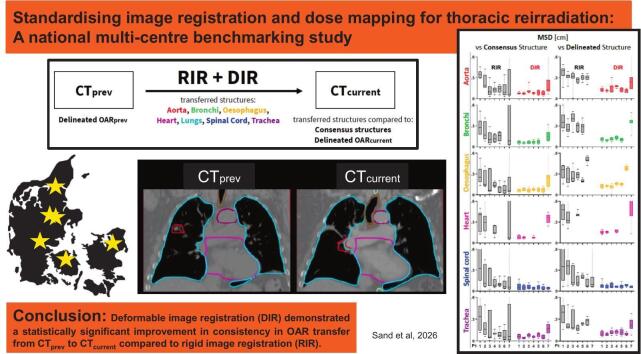

## 1 Introduction

The number of long-term survivors after thoracic cancer is increasing [1], [2], [3], [4]. However, up to one-third of lung cancer (LC) patients treated with curatively intended radiotherapy are subsequently diagnosed with locoregional recurrence or develop new LC [5], [6], [7], [8], [9], [10], [11], [12], [13], demanding effective local treatment options. Repeat high-dose radiotherapy is a promising approach and is increasingly used in clinical practice [14], [15], but associated with high toxicity risk [16], [17], [18], [19], [20], [21]. Safe delivery depends on robust evaluation of previously delivered doses on current patient anatomy. Little work has been done on standardisation and inter-centre variation of this process [22], [23], [24].

The ESTRO-EORTC consensus statement on reirradiation mandates an overlay of dose distributions in 3D for clinical work-up, including summation of equieffective doses [2]. However, a full evaluation of cumulative doses delivered across the two radiotherapy courses is often lacking in cohort studies and clinical practice due to limited access to reliable tools for accurate dose summation [3], [22]. Estimating cumulative doses requires transferring doses from previous CT image (CT_prev_) to current reirradiation CT (CT_current_), which is complicated by anatomical changes arising between treatments [25], [26], [27], [28], [29]. Variations in cumulative dose assessment across institutions can stem from differences in methodology and workflow, whether using image registration methods relying on either rigid (RIR) or deformable image registration (DIR), or simple point dose estimates [23]. Standardisation of image registration methodologies and quality assurance (QA) of registration algorithms is essential, but there is currently no consensus on optimal dose accumulation methods [30], [31], [32], [33]. RIR may often be the choice in clinical practice due to uncertainties in DIR algorithm performance [14], [24]. A single-institution study on ten locally recurrent LC patients, nevertheless, found improved accuracy for DIR [34]. A recent multi-institutional study investigating cumulative dose assessment for head-and-neck and thoracic reirradiation showed large inter-centre variations in methodology and dose assessment, with DIR yielding less variation than RIR [23]. These discrepancies affect cumulative dose accuracy, limiting the ability to link clinical toxicity to delivered dose and complicating toxicity outcome modelling.

As part of the preparation for the prospective Scandinavian CURE Lung trial (NCT06950073) of high-dose thoracic reirradiation, the Danish Oncological Lung cancer Group (DOLG) radiotherapy section conducted a national benchmarking exercise to evaluate the variability of (1) RIR and DIR and (2) dose mapping based on RIR or DIR for dose accumulation. We present the outcomes of this large multi-centre study.

## 2 Materials and methods

Seven LC cases, treated with curatively intended reirradiation near a previously irradiated region, were investigated. The cases were selected from a retrospective cohort of 74 LC patients treated with reirradiation at Aarhus University Hospital between 2013 and 2022 and were representative cases for anatomical variability seen across the cohort (supplementary material (SM) Fig. S1). For all cases, the 50% isodoses from previous and current treatments overlapped.

Organs-at-risk (OARs) were delineated on the mid-ventilation phase of both four-dimensional CT_prev_ and CT_current_ images, using artificial intelligence (AI)-assisted contouring [36], and subsequently reviewed by a senior oncologist (MK). Eight OARs were delineated: aorta, bronchi (main bronchus, intermediate bronchus, lobar bronchi), oesophagus, heart, left lung, right lung, spinal cord, and trachea. For each case, anonymised CT_prev_ and CT_current_ images, corresponding structure sets, and 3D physical dose distributions were distributed to six Danish radiotherapy centres.

### 2.1 Patient characteristics

Four females and three males were included. The median [range] interval between radiotherapy treatments was 46 months [10–85]. The median planning target volumes were 53 cm^3^ [19–697] and 54 cm^3^ [19–166] for the previous and current treatments, respectively. Four and two cases were treated with stereotactic radiotherapy in the previous and current treatments, respectively. For the remaining cases, homogeneous normo-fractionated doses were delivered in the previous treatment, and inhomogeneous dose escalation was delivered in the current treatment (further details, see Table 1).

**Table 1:** Patient characteristics. All dose prescriptions apply to the planning target volume (PTV). Fractionation schemes 30 Gy/3fx and 45 Gy/3fx are stereotactic treatments with dose escalation of the gross tumour volume (GTV) to 150%. The fractionation schemes 60 Gy/30fx and 66 Gy/33fx cover the PTV with homogeneous doses. The fractionation scheme 50 Gy/24fx includes dose escalation to the mean GTV of 65 Gy.

Patient	1	2	3	4	5	6	7
Sex	Female	Female	Female	Male	Female	Male	Male
Age	73	79	67	89	81	64	77
Previous RT							
Dose/fractions	30 Gy/3fx	30 Gy/3fx	60 Gy/30fx	30 Gy/3fx	45 Gy/3fx	60 Gy/30fx	66 Gy/33fx
Target location	Tumour: right upper lobe	Tumour: left upper lobe	Postoperative RT after pneumonectomy left, R1 resection	Tumour: right upper lobe	Tumour: left lower lobe	Postoperative RT after lobectomy right, R1 resection	Tumour: right upper lobe. Node: station 2R
PTV (cc)	29	19	212	53	45	98	697
Time between RTs (months)	55	13	46	57	85	14	10
Current RT							
Dose/fractions	66 Gy/33fx	50 Gy/24fx	40 Gy/3fx	30 Gy/3fx	50 Gy/24fx	50 Gy/24fx	50 Gy/24fx
Target location	Tumour: central/right middle lobe. Node: station 11R	Node: station 5 and left hilus	Tumour: right lower lobe	Tumour: right upper lobe	Tumour: left lower lobe	Central tumour. Node: station 2R	Node: station 2 L
PTV (cc)	79	166	19	20	54	92	26
Staging	T1bN1M0	T0N2M0	T0N0M1a	T1bN0M0	T1bN0M0	T4N2M0	T0N3M0
Histology	Adenocarcinoma	Squamous cell carcinoma	Adenoid cystic carcinoma	Squamous cell carcinoma	Squamous cell carcinoma	Squamous cell carcinoma	Squamous cell carcinoma
ReRT type	New primary	localised regional progression	Oligometastatic progression	localised regional progression	localised regional progression	Tumour recurrence	localised regional progression
PTV overlap	No	Yes	No	No	Yes	Yes	Yes
Same cc plane	Yes	Yes	Yes	Yes	Yes	Yes	Yes

### 2.2 Image registration

Image registration between CT_prev_ and CT_current_ using RIR and DIR was performed for all cases at each centre based on local guidelines (SM Table S1). The OARs were transferred from CT_prev_ to CT_current_ using both types of registrations (SM Fig. S2A). Three different software platforms were used: RayStation (one centre; version 11B [12.0.0.932]), MIM (two centres; versions 7.3.2 and 7.3.6), and Velocity (three centres: version 4.1).

For RIR, all centres used six degrees-of-freedom (6DoF) algorithms. The anatomical focus varied between centres and patients, ranging from alignment of spinal cord or chest wall to targeted registration involving the tumour and adjacent OARs on CT_current_. DIR was performed using intensity-based algorithms at all centres, with variability in the anatomical focus from whole-image registration to localised alignment around the tumour and surrounding OARs. In most cases, the DIR process was iterative, involving multiple steps until a satisfactory result was achieved. The quality of both RIR and DIR was reviewed locally by an experienced medical physicist.

To establish a common reference standard, consensus structures for each OAR, after transfer from CT_prev_ to CT_current_ via RIR and DIR, were generated on CT_current_ using the STAPLE function with a 50% agreement threshold [37] based on all transferred structures for the OAR in question. This STAPLE threshold captured the most consistent regions while limiting outlier influence. Geometric deviations between transferred and consensus structures were evaluated on CT_current_ within the clinically relevant (EQD2Gy) ≥2 Gy volume of the current plan in cranio-caudal direction, while retaining full transversal extent; this restriction was applied during preprocessing before evaluation. Similarly, deviations were evaluated between transferred structures and OAR structures delineated on CT_current_ (SM Fig. S2A). Deviations were quantified using five metrics: Dice Similarity Coefficient (DSC), Surface Dice Similarity Coefficient within 3 mm (SDSC-3 mm), Mean Surface Distance (MSD), Hausdorff distance for 98% (HD-98%), and Distance to Centre-of-Mass (DCM). Evaluations of registrations were centrally performed using CERR (MatLab, R2023a).

### 2.3 Dose mapping

Each centre mapped the 3D physical dose distribution (D_prev,phys_) from CT_prev_ to CT_current_ using RIR and DIR, respectively (SM Fig. S2B). Additionally, the dose was converted to EQD2Gy (D_prev,EQD2_) after dose mapping through the centre-specific software platform. An α/β value of 2 Gy was used for the spinal cord, with 3 Gy for the remaining seven OARs. Similarly, the dose at CT_current_ (D_current,phys_) was converted to EQD2Gy (D_current,EQD2_) at each centre, using the AI-based structures. Cumulative EQD2Gy doses (D_cum,current,EQD2_) were generated on CT_current_ by accumulating mapped and current treatment doses. Dose-volume histograms (DVHs) for mapped doses and cumulative doses for all OARs and from all centres were plotted and evaluated centrally.

### 2.4 Statistical analysis

The effect of registration method (RIR vs. DIR) on the inter-centre variation in OAR alignment between transferred OAR structures and (1) OAR structures delineated on CT_current_ and (2) consensus structures generated on CT_current_ was tested using a linear mixed-effects model, with registration method as a fixed effect and patient and centre as random effects. One model was fitted for each OAR and image registration metric. Similarly, the effect of registration method on the inter-centre variation in the accumulated dose on CT_current_ for each OAR was tested for (1) OAR dose metrics corresponding to key constraints values from the CURE Lung protocol [35] evaluated on D_cum,current,EQD2_, and (2) the difference in DVH area, defined as the interquartile range (IQR) of the DVH curves across centres at all dose points, sampled at 0.1 Gy intervals. For both datasets, paired RIR and DIR values for individual patients were plotted to identify whether extremes in the distributions were driven by single patients. While the area under a cumulative DVH-curve equals the mean dose, the band area quantifies inter-center variability across all dose levels, representing overall DVH dispersion rather than a single metric. For all statistical analyses, a two-sided significance level of 5% was used.

### 2.5 Ethics

Permission to access patient journals, scans, and treatment planning material for retrospective research was obtained from the Central Region of Denmark.

## 3 Results

**Fig. 1:**
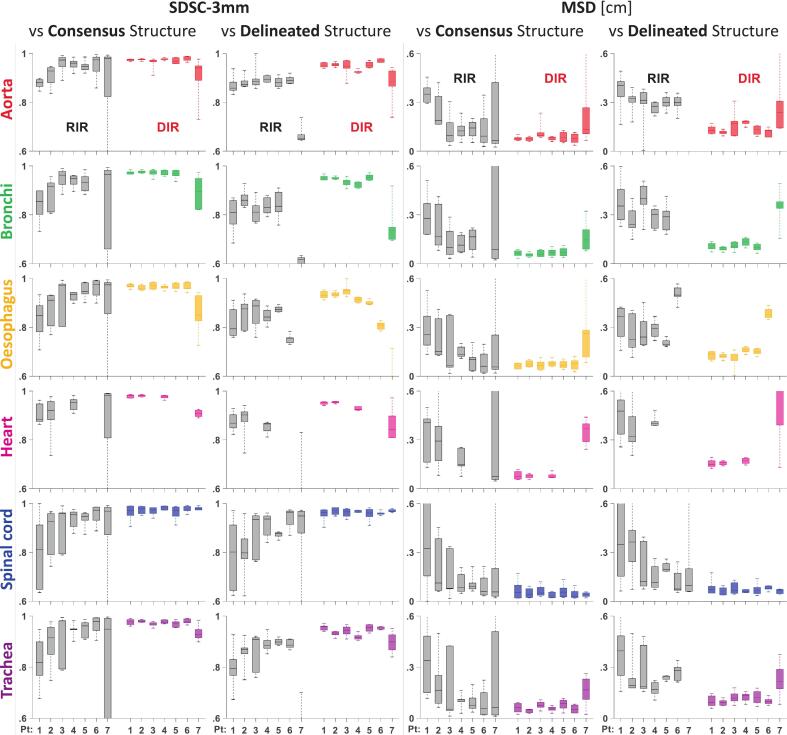
Inter-centre geometric variation for structures transferred from CT_prev_ to CT_current_ using RIR (grey) and DIR (coloured), compared to the consensus structures and the delineated OAR structures on CT_current_, assessed by the Surface Dice Similarity Coefficient within 3 mm (SDSC-3 mm) and the Mean Surface Distance (MSD). Median and the 25% and 75% percentiles and outliers are shown.

**Fig. 2:**
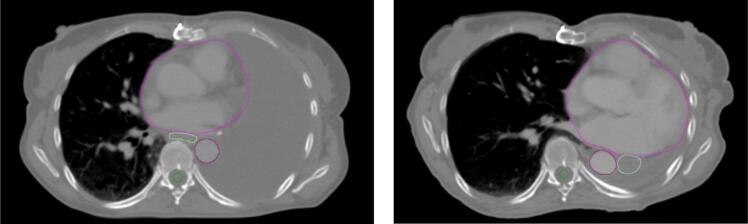
Major anatomical changes occurred for case 7 between CT_prev_ and CT_current_, due to the appearance of an atelectasis, resulting in geometrical misalignment of the OARs for both RIR and DIR. The heart (purple), oesophagus (white), aorta (red), and spinal cord (green) are delineated.

Seven cases were evaluated with both RIR and DIR at six centres, corresponding to 84 unique image registrations. Fig. 1 illustrates the geometric variation among centres and registration methods for the SDSC-3 mm and MSD metrics. The geometric deviations between transferred OARs and consensus OARs or delineated OARs are both presented, and only minor differences were seen. DIR reduced the geometric deviation between individually registered OARs from each centre to both consensus and delineated OARs, compared to RIR. SDSC-3 mm values were statistically significantly higher (p ≤ 0.05 in mixed-effects modelling) and MSD values were statistically significantly lower (p ≤ 0.05) for DIR than for RIR across all OARs, for individual organs, and for patients 1–6 (Figs. 1, S3-S4, Table S2). For DSC, HD-98%, and DCM, see Figs. S3-6, Table S2). Results for cases 1–6, SDSC-3 mm > 0.78 and MSD < 4.4 mm were observed among all boxplots when using DIR, with lower consistency for case 7, where major anatomical changes occurred between CT_prev_ and CT_current_, due to the appearance of an atelectasis, resulting in geometrical misalignment of the OARs for both RIR and DIR (Fig. 2). Consequently, we excluded case 7 from the dose evaluations, as proceeding with image registration-based dose mapping in a real clinical scenario would have been deemed irresponsible.

Rescaling of D_current,phys_ to D_current,EQD2_ on CT_current_ resulted in identical DVH-bands across all centres, demonstrating independence of EQD2 rescaling of centre-specific software programs used (SM Fig. S7).

**Fig. 3:**
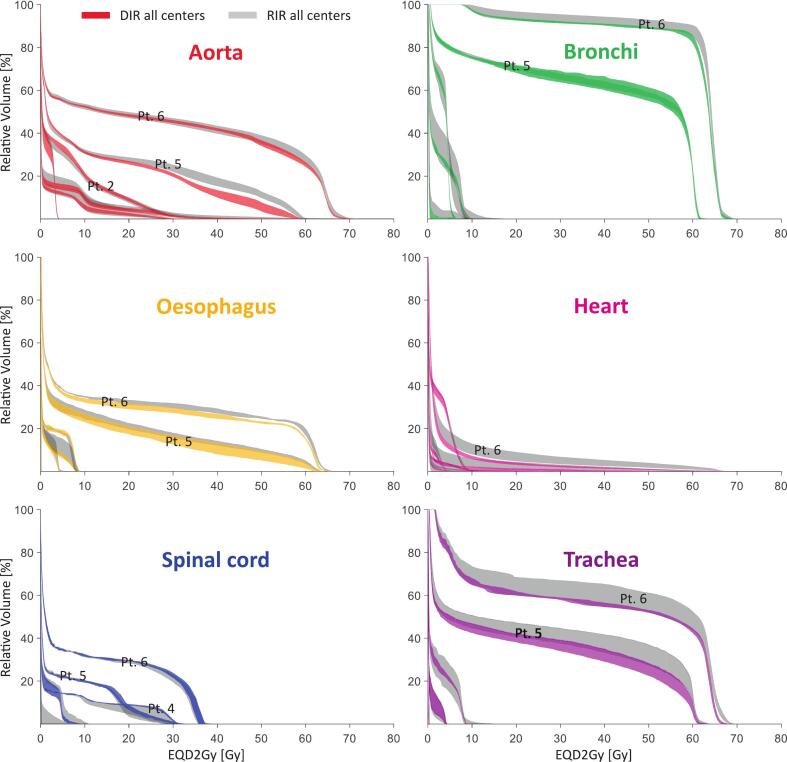
. DVH bands for six OARs for cases (pt) 1–6, illustrating the minimum to maximum values across all centres for both RIR (grey) and DIR (coloured) for the mapped 3D doses at CT_current_ converted to EQD2Gy(D_pre,EQD2_).

Fig. 3 presents DVH-bands for six OARs for cases 1–6, illustrating minimum to maximum values across all centres for both RIR and DIR for D_pre,EQD2_ (for D_cum,curr,EQD2_ and lungs see Figs. S8-S9). A substantial inter-centre and inter-patient variation was found for both RIR and DIR (Figs. 3 and S10).

**Fig. 4:**
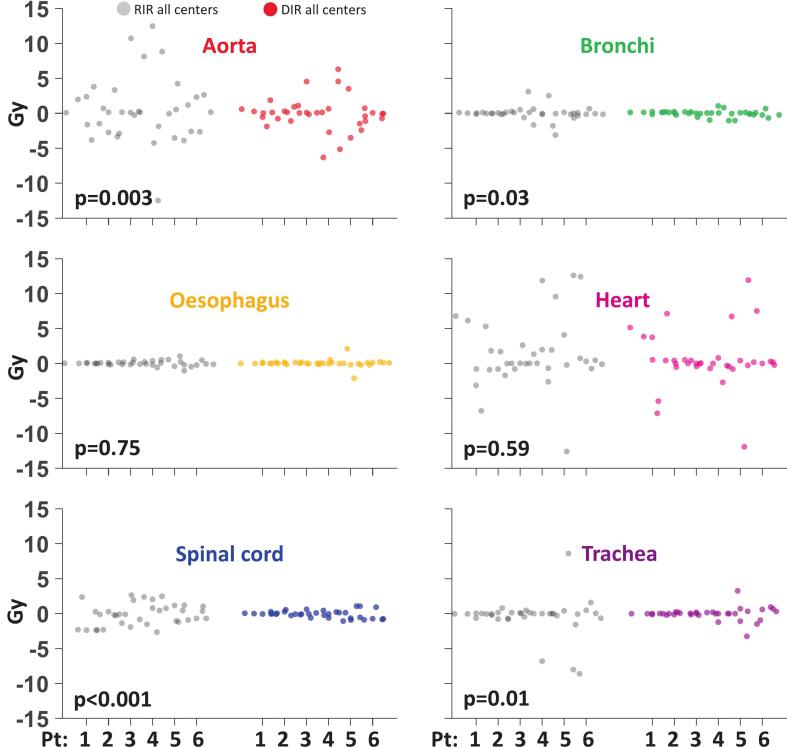
Swarm charts for six OARs for cases (pt) 1–6, illustrating inter-centre variation symmetrised around 0 Gy for RIR (grey) and DIR (coloured), for dose-maximum constraints (D_0.3cc_) for the cumulated EQD2Gy doses (D_cum,curr,EQD2_). For each OAR p-values are quoted.

Fig. 4 presents all centre values [minimum–maximum, symmetrized around 0 Gy] for RIR and DIR for a selection of priority 1 and 2 dose-maximum constraints (D_0.3cm_^3^) for D_cum,curr,EQD2_ to the OARs, mandatory to comply with in the CURE Lung trial and prioritised over target dose coverage. For all constraints, DIR demonstrated significantly less variability than RIR (p < 0.05), except for oesophagus (p = 0.75) and heart (p = 0.59). Analysis of the area within the interquartile range of the full DVH-bands (see Figs. 3 and S9) demonstrated no statistically significant difference in the variation of the mapped dose distributions between RIR and DIR for all OARs, except for the bronchi (p = 0.032) for both D_prev,EQD2_ and D_cum,curr,EQD2_, and for aorta (p = 0.023) for D_cum,curr,EQD2_, both showing a statistically significant smaller area with DIR. DVH-band areas and p-values are shown in Table S3.

## 4 Discussion

The increasing number of re-irradiated patients mandates the establishment of best practices for image registration and dose accumulation. Understanding inter-centre consistency and performance with modern clinical software systems is a key step. This is the first large-scale multi-centre study focusing on comparison and consistency of image registration methodologies for dose mapping for reirradiation. We have demonstrated that DIR provides statistically significant better consistency in transferring OARs from CT_prev_ to CT_current_ than RIR. For the DVHs, significantly less inter-centre variations were observed with DIR compared to RIR. However, no significant difference was found for the overall DVH curves, evaluated as the area between the interquartile ranges of DVH curves.

Two smaller-scale studies have reported results consistent with our findings. The multi-centre dose accumulation study conducted by Hardcastle et al. included one LC case and compared cumulative doses obtained after RIR and DIR in 12 centres [23]. They found less variation after using DIR compared to RIR. This finding aligns with a single-institutional study on ten LC patients, where the registration accuracy for DIR improved by 3.0 mm compared to RIR in a surface-based comparison of transferred OAR structures to manually delineated structures on CT_current_ [34]. In the current study, the geometrical difference between transferred OARs was significantly reduced by DIR. In most cases, MSD was below 3 mm for all OARs, except for case 7, which had large anatomical changes between treatment courses.

Inter-centre variation in image registration primarily originates from two sources: 1) the use of different DIR algorithms and their varying software system implementation, and 2) different image registration workflows and preferences, which impact both RIR and DIR. Previous work on DIR in the on-treatment dose accumulation setting has demonstrated significant impact of the choice of DIR algorithm on mapped dose [27], [38]; but with less variation for intensity-based algorithms (employed by all centres in the current study) [38], and with the uncertainty associated with different DIR algorithms less than that of anatomical changes occurring during the radiotherapy course [27]. Although not a formal part of the study aims, we did not observe any dependence on image registration software systems, suggesting that the likely main source of variation was user choices and workflows. Especially the region of interest (ROI) choice impacts the registration outcome, and this may be particularly important for RIR, where small changes in the registration ROI can affect global translations and rotations [23]. We generally observed more inter-centre variation for RIR (D_0.3cm_^3^, 1–25 Gy) compared to DIR (1–12 Gy), supporting the hypothesis of variation being primarily user-driven.

Reirradiation patients often exhibit considerable anatomical deformations between treatment courses, supporting the use of DIR over RIR. The geometrical uncertainty may be difficult to quantify and validate for individual patients, which has led to preferences for RIR in many clinics [14], [31], despite the superior reported performance of DIR [23], [34], [39]. The geometrical uncertainties will manifest in uncertainties in the transferred doses [25], but the specifics will be highly dependent on the dose distribution. In regions with steep dose gradients, a low point-to-point error is essential, whereas in regions with homogeneous doses, larger point-to-point errors will not affect the mapped dose [25], [40], [41]. This was seen in case 4, where the PTVs of previous and current targets were overlapping and located close to the aorta, leading to steep dose gradients towards the aorta for both plans. This resulted in inter-centre dose variations in cumulative D_0.3cm_^3^ of 13 and 25 Gy for DIR and RIR, respectively, underpinning that even for closely located targets, large inter-centre dosimetric variations can be observed for nearby OARs. For case 7, neither DIR nor RIR resulted in a clinically acceptable transfer of the OARs and no dosimetric evaluation was performed. In such extreme cases, simple near-maximum point dose assessments for each OAR obtained by multiple RIRs with different ROIs may be the only clinically acceptable option [25], [32], [42].

Image registration QA is strongly recommended in the AAPM TG 132 report [30]. It is not standardised, especially not for dose accumulation purposes [3], [22], [29], [31], [33]. A variety of quantitative methods exists, based on geometric measures e.g. point-based metrics using landmarks visualised on paired images, evaluation of local volume change resulting from the registration by the Jacobian determinant, directional independence (inverse consistency), image similarity, and deformation vector field visualisation [30], [33]. As a bare minimum, a visual inspection must be performed. For our work, experienced physicists performed this check for each case as part of the standard workflows. A limitation of this study is the use of contour-based metrics alone. Including anatomical landmarks such as blood vessels or airway bifurcations [43], could have provided additional information on registration accuracy beyond structure boundaries, particularly for large organs like the lungs.

In the current study, the OARs were delineated on CT_prev_ and CT_current_ using AI-based contouring [36] and reviewed by an experienced oncologist. This ensured standardised delineation, making geometrical comparison of the structures transferred from CT_prev_ to CT_current_ reliable. Two methods were used to assess geometric and volumetric differences between OARs transferred and OARs delineated on CT_current_: 1) comparison to delineated structures and 2) comparison to consensus structures created using the STAPLE function. A slightly better performance was observed for the comparison to the STAPLE function; indicating either a consistent offset in image registration or differences in OAR delineation between treatment courses, despite the use of auto-contouring. For the heart, auto-contouring was less reliable superiorly and inferiorly, due to low soft tissue contrast.

While equieffective dose accumulation is recommended [2], the cumulative equieffective dose is highly affected by the choice of α/β value(s) and potential tissue recovery [23]. As there is no consensus on organ-specific α/β values and tissue recovery, a fixed value of 2 Gy for spinal cord and 3 Gy for all other OARs was selected to mimic the biological difference between nerves and soft tissues [21], [23]. This will result in an artificial step in the dose distribution. However, in clinical practice, only doses to individual OARs will be evaluated and used in the planning process. All software systems used in this study handled the calculation of equieffective dose identically, with variability between systems arising only from dose mapping.

The number of patients who could benefit from reirradiation is increasing, which raises the need for clinical reirradiation guidelines [1], [2], [3], [24]. The treatment planning strategy often balances full target coverage and avoiding OAR over-dosage, and inaccurate dose assessment may lead to severe target under-dosage or OAR over-dosage. Standardised approaches are therefore essential to ensure consistent patient management in multi-centre settings. Additionally, cumulative dose constraints are largely based on clinical experience and established through consensus strategies [21]; changing this will require high-quality cumulative dose data in combination with prospectively collected toxicity data [15].

This study was made in preparation for the prospective Scandinavian CURE Lung trial on reirradiation for LC patients. The trial aims to deliver safe curative reirradiation for thoracic cancers based on standardised image registration, dose planning and clinical follow-up to increase the number of patients offered curative reirradiation. Based on this pre-trial benchmarking study, the protocol recommends using DIR except for cases with large anatomical changes. A trial QA group will supervise centres on image registration in complex cases and perform on-trial QA to ensure high compliance with trial procedures. The standardisation and continuous QA surveillance ensure that data can be used to model the impact of cumulative dose levels on clinical toxicity after reirradiation. The trial aims for 500 patients in five years.

In conclusion, DIR demonstrated a statistically significant improvement in consistency in OAR transfer from CT_prev_ to CT_current_ compared to RIR. For near-maximum doses, significantly less variation was seen for DIR. This supports the preferential use of DIR for reirradiation planning and cumulative dose evaluation in LC and will be crucial for ensuring reproducible and safe reirradiation in the forthcoming CURE Lung trial. However, inter-centre dosimetric variations may still be substantial in regions with steep dose gradients.

## CRediT authorship contribution statement

**Hella Sand:** Methodology, Software, Formal analysis, Resources, Writing – original draft, Writing – review & editing, Visualization. **Dennis Arp:** Methodology, Formal analysis, Writing – review & editing. **Maria F Jensen:** Methodology, Formal analysis, Writing – review & editing. **Marianne M Knap:** Methodology, Formal analysis, Writing – review & editing. **Christina Larsen:** Methodology, Formal analysis, Writing – review & editing. **Mikkel D Lund:** Methodology, Formal analysis, Writing – review & editing. **Morten Nielsen:** Methodology, Formal analysis, Writing – review & editing. **Wiviann Ottosson:** Methodology, Formal analysis, Writing – review & editing. **Ane Appelt:** Conceptualization, Methodology, Formal analysis, Resources, Writing – original draft, Writing – review & editing, Visualization, Supervision, Project administration. **Lone Hoffmann:** Conceptualization, Methodology, Formal analysis, Resources, Writing – original draft, Writing – review & editing, Visualization, Supervision, Project administration.

## Declaration of competing interest

The authors declare the following financial interests/personal relationships which may be considered as potential competing interests: The project was supported by the Danish Cancer Society [grants no. R374-A22286 and R302-A17275], and the Danish Comprehensive Cancer Center. AA acknowledges Cancer Research UK funding for the Leeds Radiotherapy Research Centre of Excellence (RRCOER-Jun24/100004).
