# Supplementary material for: Standardising image registration and dose mapping for thoracic reirradiation: A national multi-centre benchmarking study
Source: Phys Imaging Radiat Oncol. 2026 Jan 29;37:100913. doi: 10.1016/j.phro.2026.100913 (PMC12886061; doi:10.1016/j.phro.2026.100913)
Supplement: Supplementary Data 1 [file mmc1.pdf]

Supplementary Fig. S1: Representative CT images from the initial (pre-RT) and re-irradiation (reRT) scans for all seven patients, shown as transverse, sagittal, and frontal slices. Delineation of the GTV is shown in red, the lungs are shown in turquoise, the heart is shown in pink, the trachea and bronchi are shown in brown, the spinal cord is shown in green, and the esophagus is shown in light green.

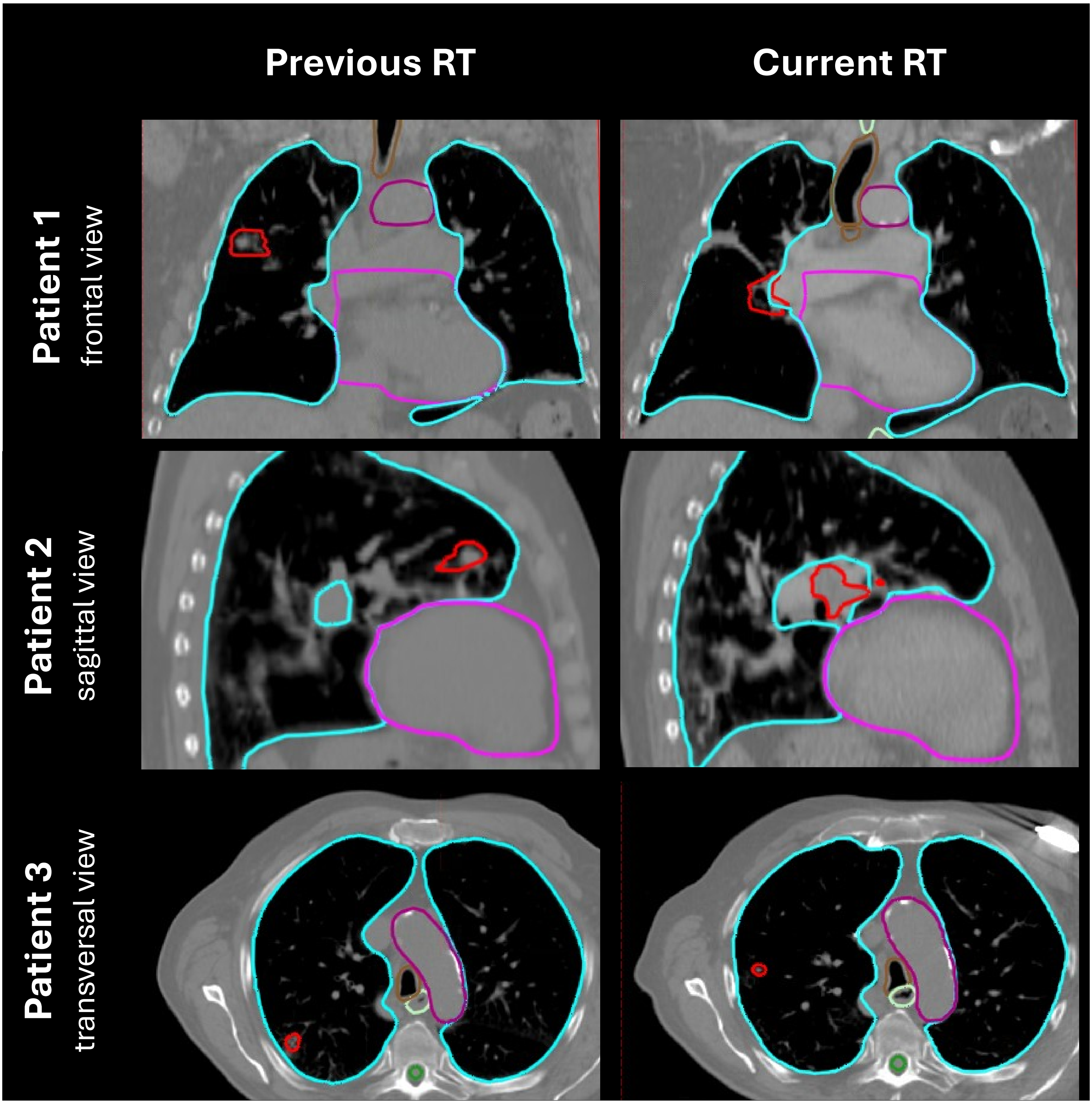

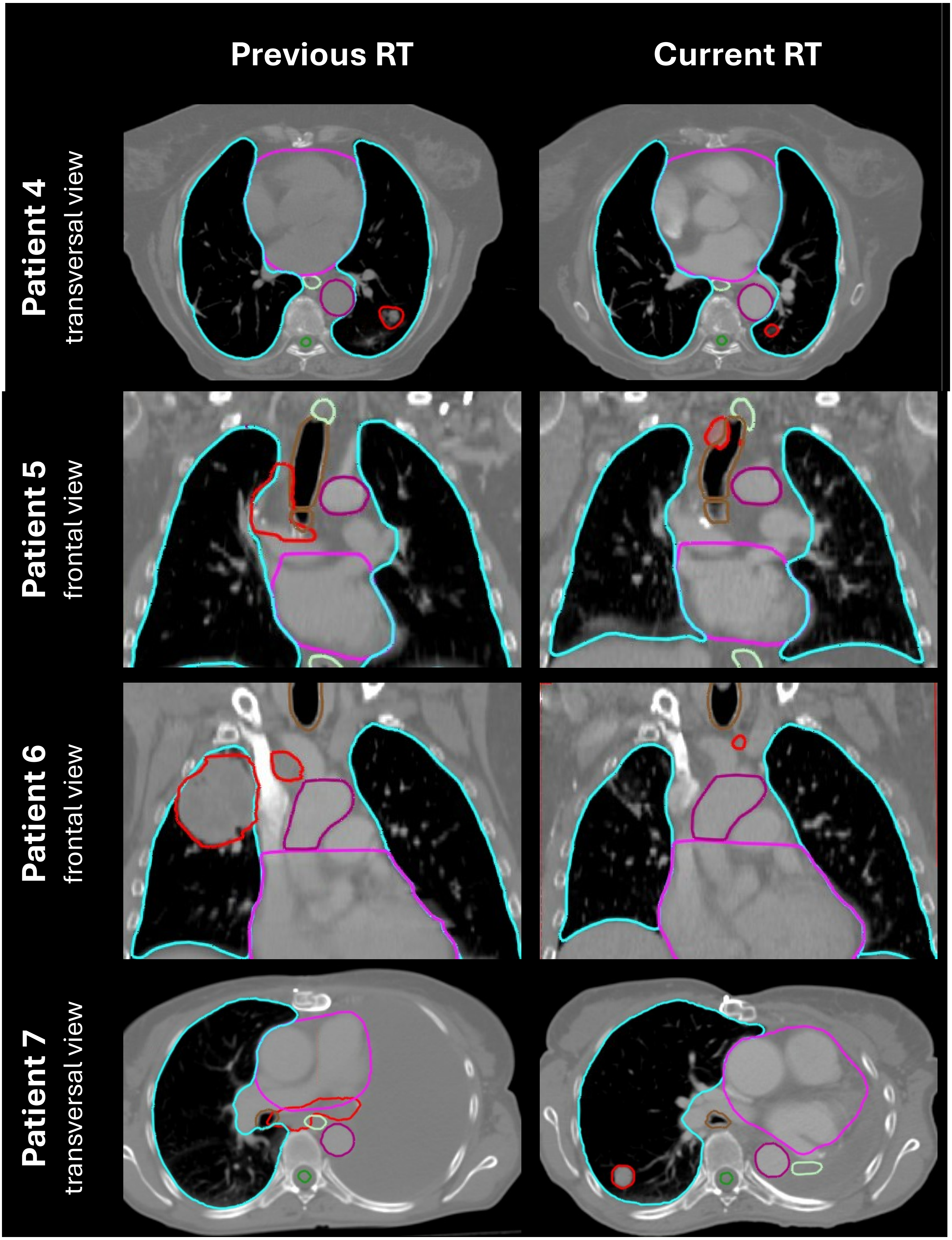
Previous RT
Current RT
Patient 4
transversal view
Patient 5
frontal view
Patient 6
frontal view
Patient 7
transversal view

Supplementary Table S1. Clinical workflows for image registraion for the three different software solutions.

In Velocity:

Step 1 Rigid image registration (RIR) from CT_{prev} to $CT_{current}$. The region of interest (ROI) and the origin of registration vary between clinics and cases. Six degrees of freedom are always used.
Step 2a Deformable image registration (DIR) from CT_{prev} to $CT_{current}$ for the entire image.
Step 2b Further optimizing DIR on top of Step 2a: DIR from CT_{prev} to $CT_{current}$ in a ROI covering the target area on $CT_{current}$ with a 10 cm margin.
Step 2c Further optimizing DIR on top of Step 2b: DIR from CT_{prev} to $CT_{current}$ with special focus on critical OARs near the target on $CT_{current}$.
Step 3 Visual verification of the DIR in a clinically relevant area, with special focus on critical OARs near the target, performed by an experienced physicist.
Step 4 Transfer structures and doses from CT_{prev} to $CT_{current}$.
Step 5 Select α/β values for the OARs
Step 6 Calculate equieffective (EQD2Gy) doses for both dose distributions
Step 7 Accumulate equieffective doses on $CT_{current}$.

In MIM:

Step 1 Rigid image registration (RIR) from CT_{prev} to $CT_{current}$. The origin of the registration is located near the current tumour. The region of interest (ROI) vary between clinics and cases. Six degrees of freedom are always used.
Step 2a Deformable image registration (DIR) from CT_{prev} to $CT_{current}$ for the entire image.
Step 2b If needed: further optimizing DIR on top of Step 2a using a ROI covering the target and relevant OARs on $CT_{current}$.
Step 3 Visual verification of the DIR in a clinically relevant area, with special focus on critical OARs near the target, performed by an experienced physicist.
Step 4 Transfer structures and doses from CT_{prev} to $CT_{current}$.
Step 5 Select α/β values for the OARs
Step 6 Calculate equieffective (EQD2Gy) doses for both dose distributions
Step 7 Accumulate equieffective doses on $CT_{current}$.

In RayStation:

Step 1 Rigid image registration (RIR) from CT_{prev} to $CT_{current}$. The region of interest (ROI) and the origin of registration vary between cases. Six degrees of freedom are always used.
Step 2a Deformable image registration (DIR) from CT_{prev} to $CT_{current}$ for the entire image using the ANACONDA algorithm with default settings. $CT_{current}$ used as Reference Image, CT_{prev} as Target image.
Step 2b If needed, relevant ROIs (e.g. Lungs and Chest Wall) were used to guide the DIR as so-called Focus ROIs. This was chosen on a case-by-case basis, and only needed in one out of six cases.
Step 3 Visual verification of the DIR in a clinically relevant area, with special focus on critical OARs near the target, performed by an experienced physicist.
Step 4 Transfer structures and doses from CT_{prev} to $CT_{current}$.
Step 5 Select α/β values for the OARs
Step 6 Calculate equieffective (EQD2Gy) doses for both dose distributions
Step 7 Accumulate equieffective doses on $CT_{current}$.

Supplementary Fig. S2A+B. Image registration and dose mapping between CT_{prev} and $CT_{current}$ using RIR and DIR. Upper panel: Delineated OARs (OAR_{prev}) were transferred from CT_{prev} to $CT_{current}$ using DIR and RIR, and compared to both consensus structures and delineated OAR ($OAR_{current}$) structures on $CT_{current}$. Lower panel: The 3D physical dose distribution ($D_{prev,phys}$) was mapped from CT_{prev} to $CT_{current}$ using RIR and DIR. On $CT_{current}$, the dose was converted to EQD2Gy ($D_{prev,EQD2}$). The dose at $CT_{current}$ ($D_{current,phys}$) was converted to EQD2Gy dose ($D_{current,EQD2}$). Cumulative EQD2Gy doses ($D_{cum,current,EQD2}$) were generated on $CT_{current}$ by accumulating mapped and current treatment doses.

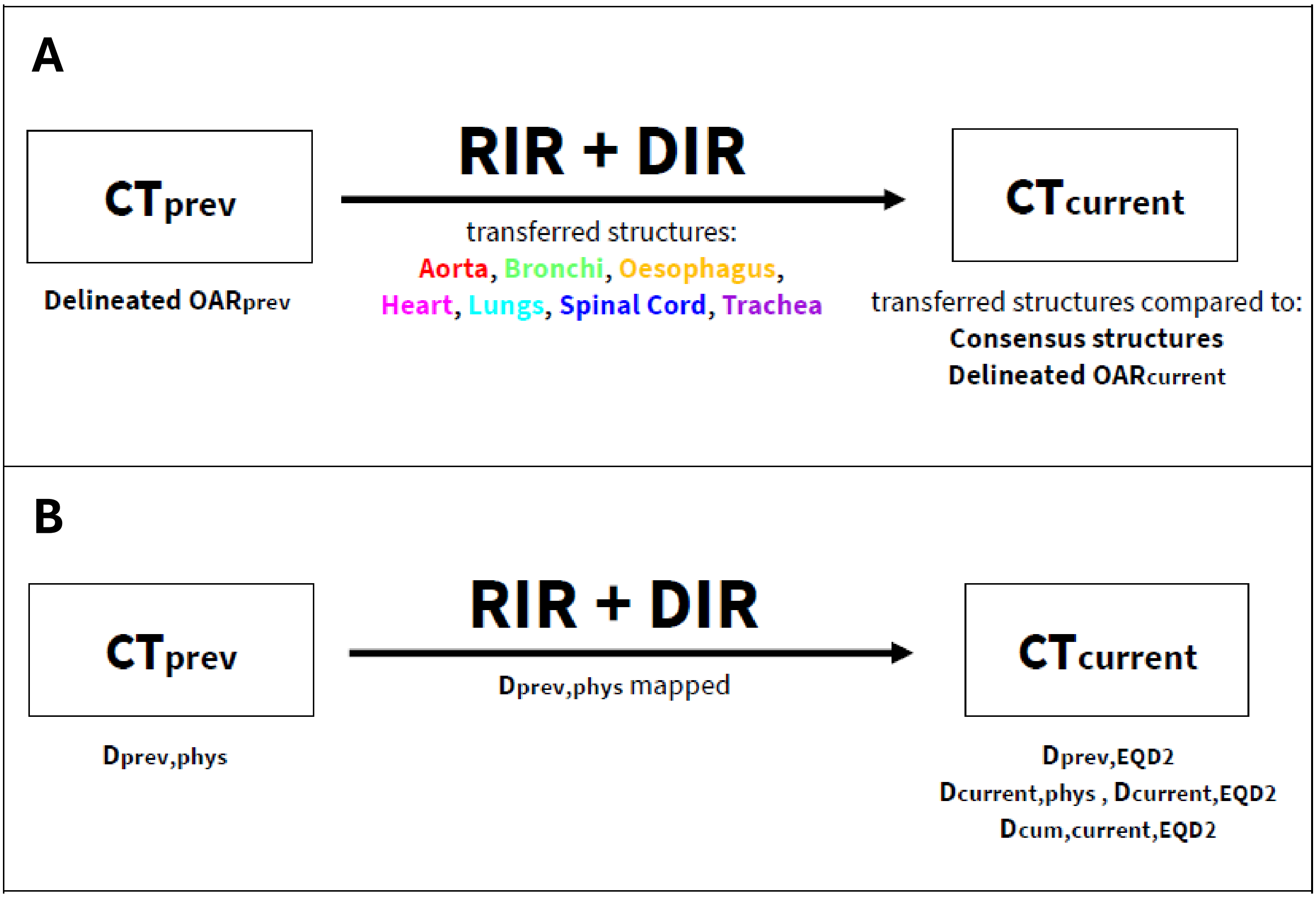

Supplementary Table S2. Statistical analyses of DIR vs. RIR for each OAR and all OARs transferred from CT_{prev} to $CT_{current}$.

		Consensus	Delineated			Consensus	Delineated
**Metric**	**OAR**	**p_value**	**p_value**	**Metric**	**OAR**	**p_value**	**p_value**
SDSC-3mm	Aorta	0,0056	0,0000	SDSC-3mm	Bronchi	0,0018	0,0000
MSD		0,0076	0,0000	MSD		0,0026	0,0000
DSC		0,0026	0,0000	DSC		0,0002	0,0000
HD-98%		*0,3721*	0,0016	HD-98%		0,0096	0,0000
DCM		*0,1004*	0,0000	DCM		0,0008	0,0000

Metric	OAR	p_value	p_value	Metric	OAR	p_value	p_value
SDSC-3mm	Oesophagus	0,0102	0,0000	SDSC-3mm	Heart	0,0122	0,0000
MSD		0,0332	0,0000	MSD		0,0237	0,0000
DSC		0,0009	0,0000	DSC		0,0226	0,0000
HD-98%		*0,1888*	0,0029	HD-98%		*0,5097*	0,0321
DCM		*0,0955*	0,0001	DCM		0,0082	0,0000

Metric	OAR	p_value	p_value	Metric	OAR	p_value	p_value
SDSC-3mm	Spinal cord	0,0004	0,0000	SDSC-3mm	Trachea	0,0028	0,0000
MSD		0,0056	0,0010	MSD		0,0051	0,0000
DSC		0,0000	0,0000	DSC		0,0006	0,0000
HD-98%		0,0002	0,0000	HD-98%		0,0015	0,0000
DCM		0,0002	0,0000	DCM		0,0004	0,0000

Metric	OAR	p_value	p_value	Metric	OAR	p_value	p_value
SDSC-3mm	LungLeft	0,0000	0,0000	SDSC-3mm	LungRight	0,0013	0,0000
MSD		0,0000	0,0000	MSD		0,0030	0,0000
DSC		0,0000	0,0000	DSC		0,0017	0,0000
HD-98%		0,0000	0,0000	HD-98%		0,0117	0,0000
DCM		0,0000	0,0000	DCM		0,0004	0,0000

Metric	OAR	p_value	p_value
SDSC-3mm	All	0,0000	0,0000
MSD		0,0000	0,0000
DSC		0,0000	0,0000
HD-98%		0,0000	0,0000
DCM		0,0000	0,0000

Consensus applies to the individually registered OARs compared to the consensus structures. **Delineated** applies to the individually registered OARs compare to the delineated OARs. Linear mixed-effects model were used, with registration method as a fixed effect and patient and centre as random effects. The p_values measures two-sided 5% significance between RIR and DIR. P-values in italic are not significant.

Supplementary Fig. S3. Inter-centre geometric variation for structures transferred from CT_{prev} to $CT_{current}$ using RIR (grey) and DIR (coloured), compared to the consensus structures (the STAPLE function with a 50% agreement threshold) and the delineated OAR structures on $CT_{current}$, assessed by the Dice Similarity Coefficient (DSC) and the Surface Dice Similarity Coefficient within 3 mm (SDSC-3mm).

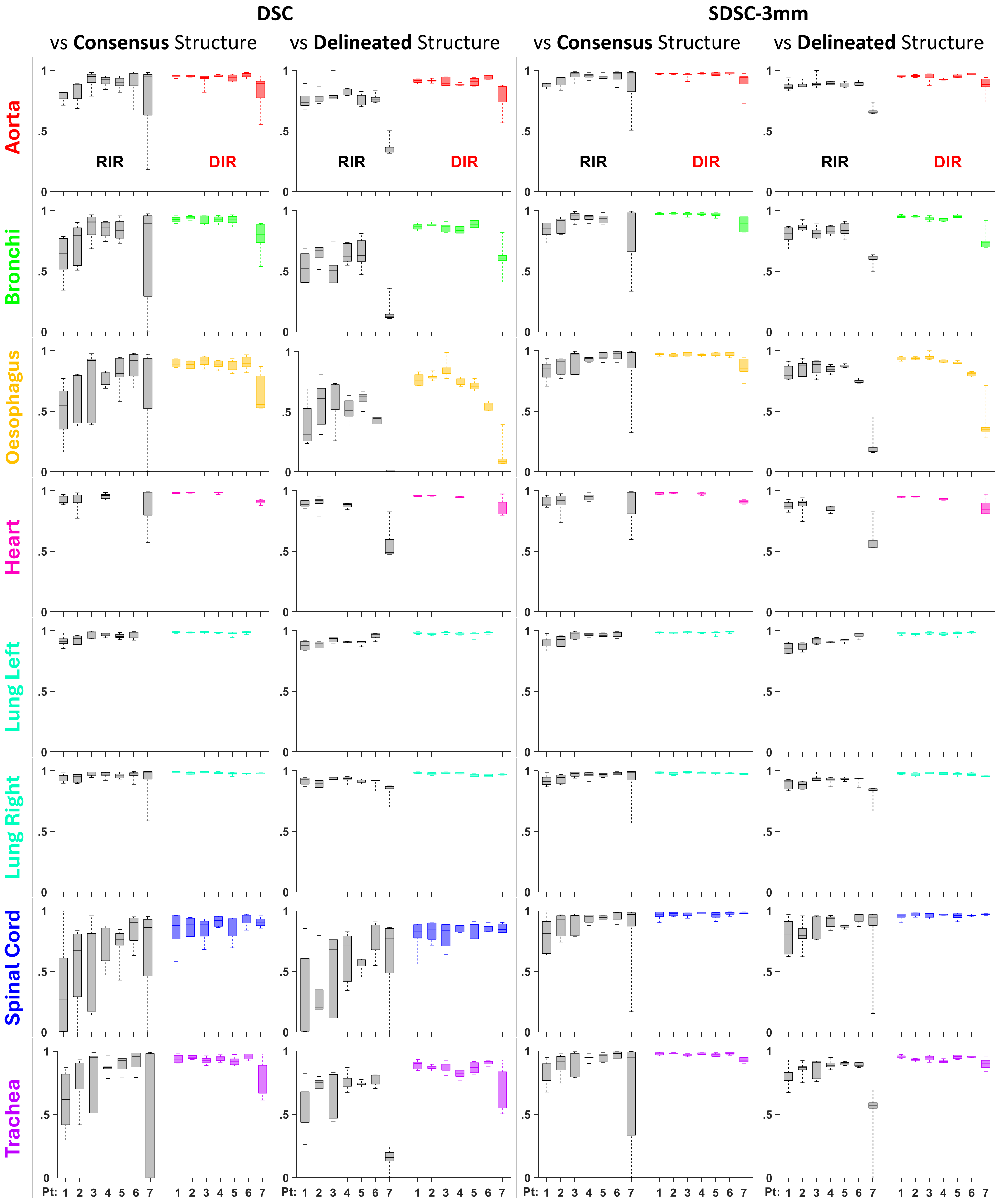

Supplementary Fig. S4. Inter-centre geometric variation for structures transferred from CT_{prev} to $CT_{current}$ using RIR (grey) and DIR (coloured), compared to the consensus structures (the STAPLE function with a 50% agreement threshold) and the delineated OAR structures on $CT_{current}$, assessed by the Mean Surface Distance (MSD) and the Hausdorff distance for 98% of the points (HD-98%).

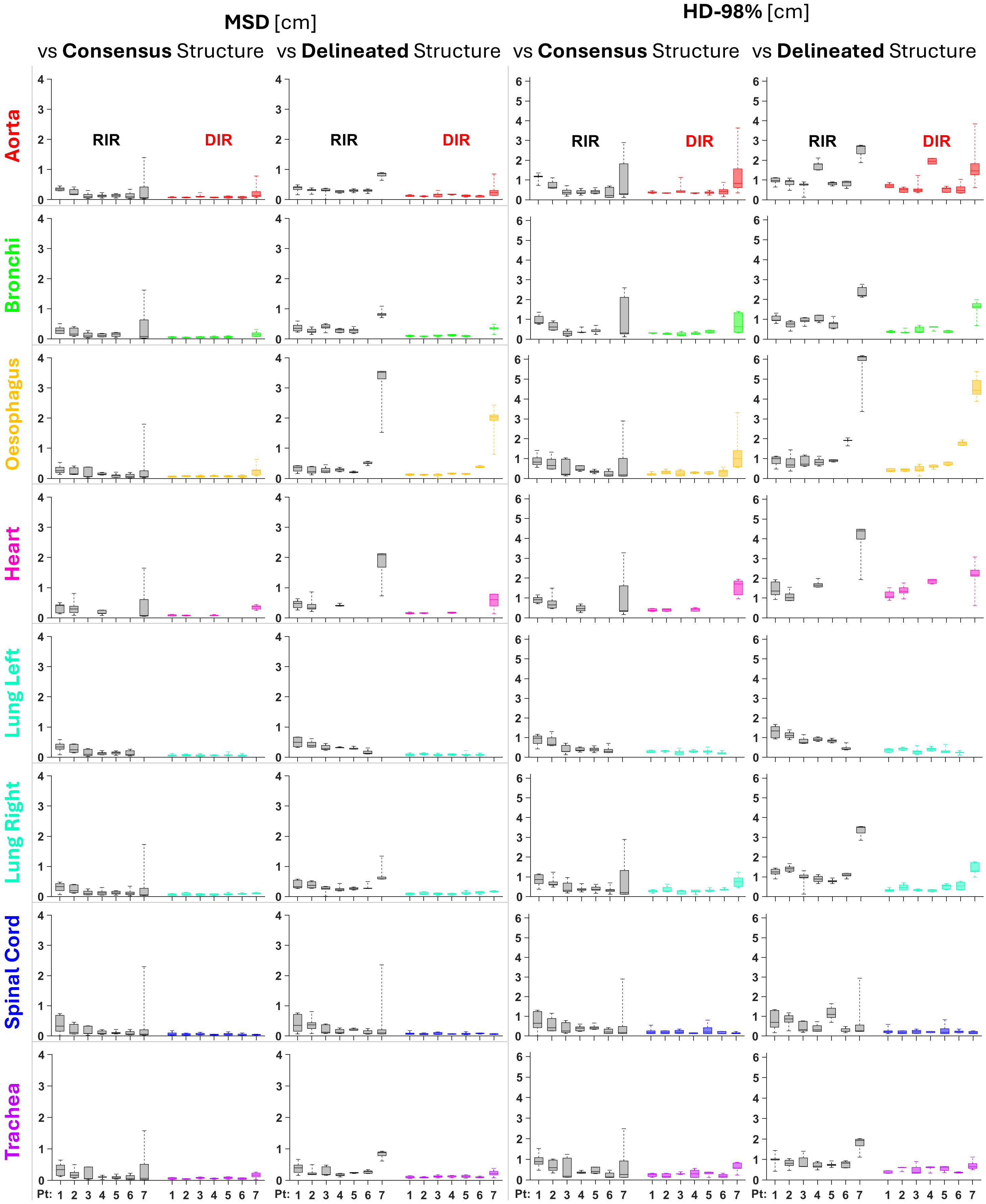

Supplementary Fig. S5. Inter-centre geometric variation for structures transferred from CT_{prev} to $CT_{current}$ using RIR (grey) and DIR (coloured), compared to the consensus structures (the STAPLE function with a 50% agreement threshold) and the delineated OAR structures on $CT_{current}$, assessed by the Distance to Centre of Mass (DCM).

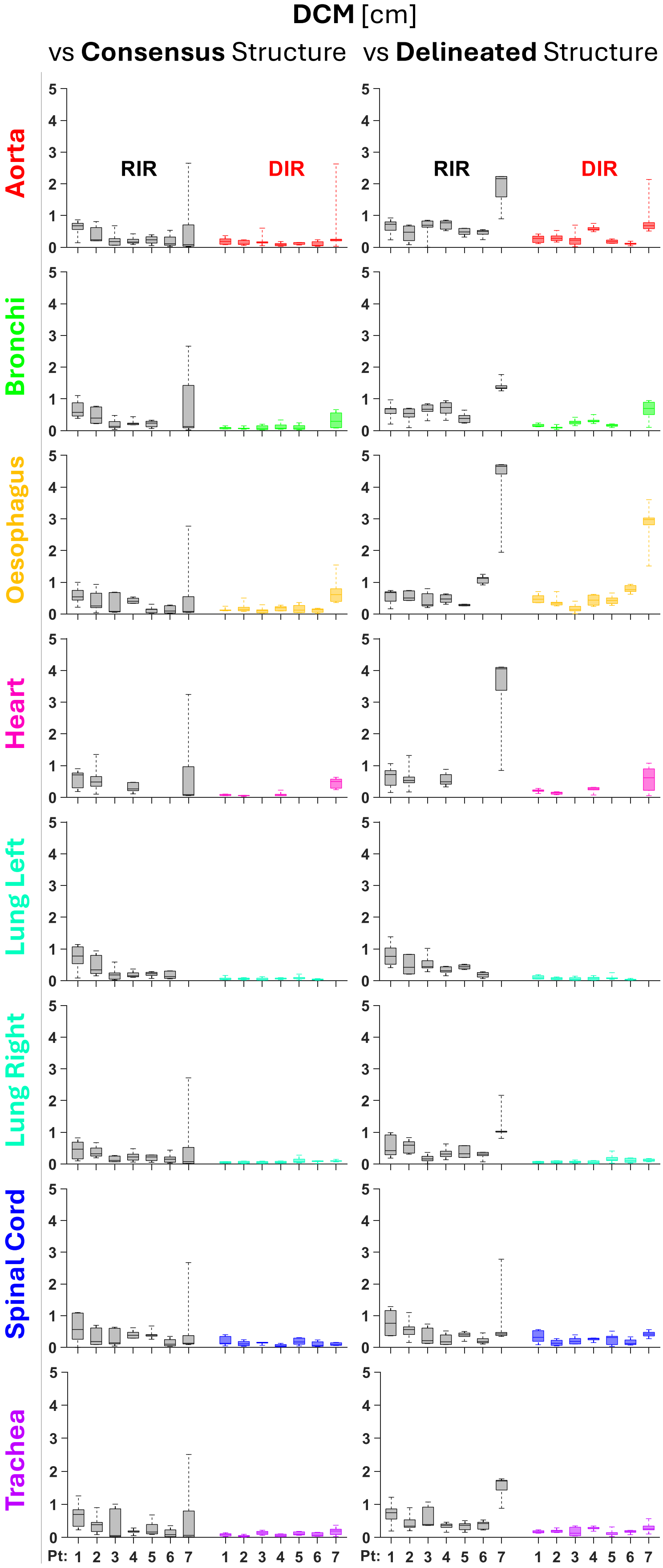

Supplementary Fig. S6. Unity plots of the paired RIR and DIR values for individual patients and OAR corresponding to the data in Fig. 1.

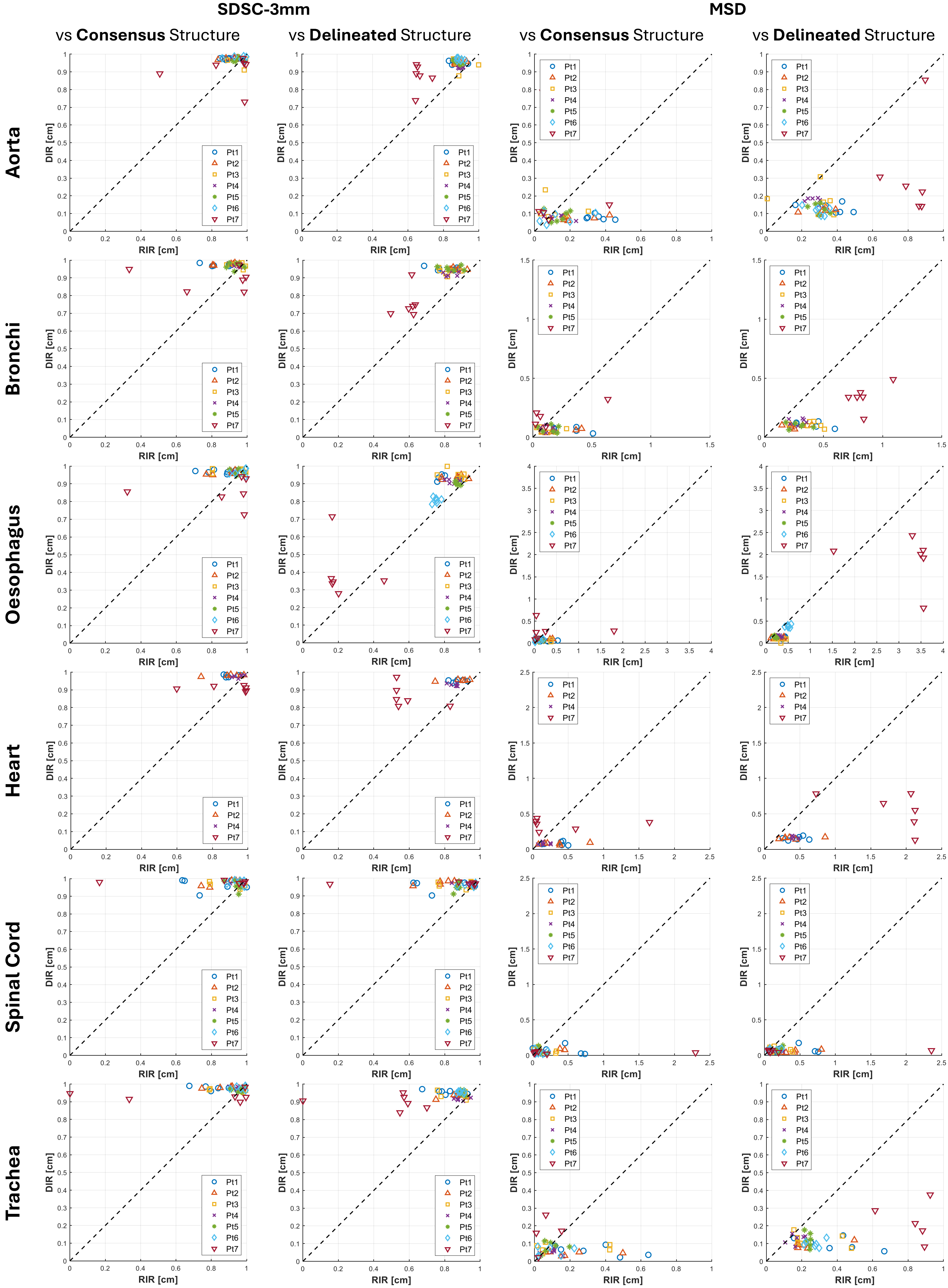

Supplementary Fig. S7. DVH bands for the bronchi structure from (pt) case 2, showing six overlaid DVH curves (one from each centre) after rescaling from $D_{current,phys}$ to $D_{current,EQD2}$ on $CT_{current}$. The complete overlap of the DVH bands illustrates consistency across all centres, regardless of local software implementations. Identical results were observed for all otherOARs across all patients.

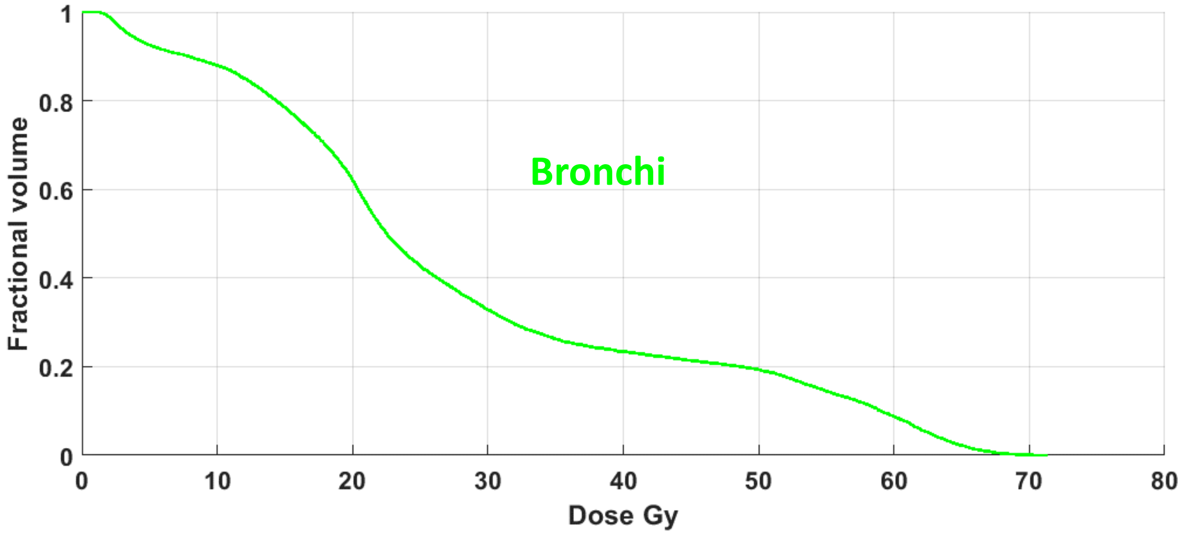

Supplementary Fig. S8. DVH bands for Lung Left and Lung Right across all cases, showing the minimum to maximum values across all centres for both RIR and DIR, for the doses **(A)** $D_{pre,EQD2}$ and **(B)** $D_{cum,curr,EQD2}$,

A

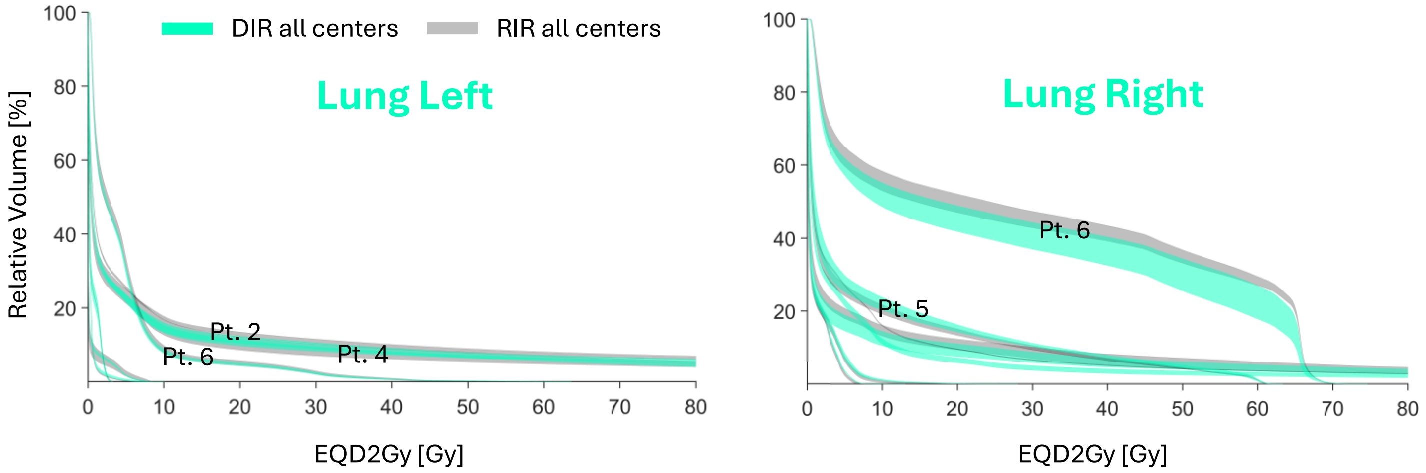

B

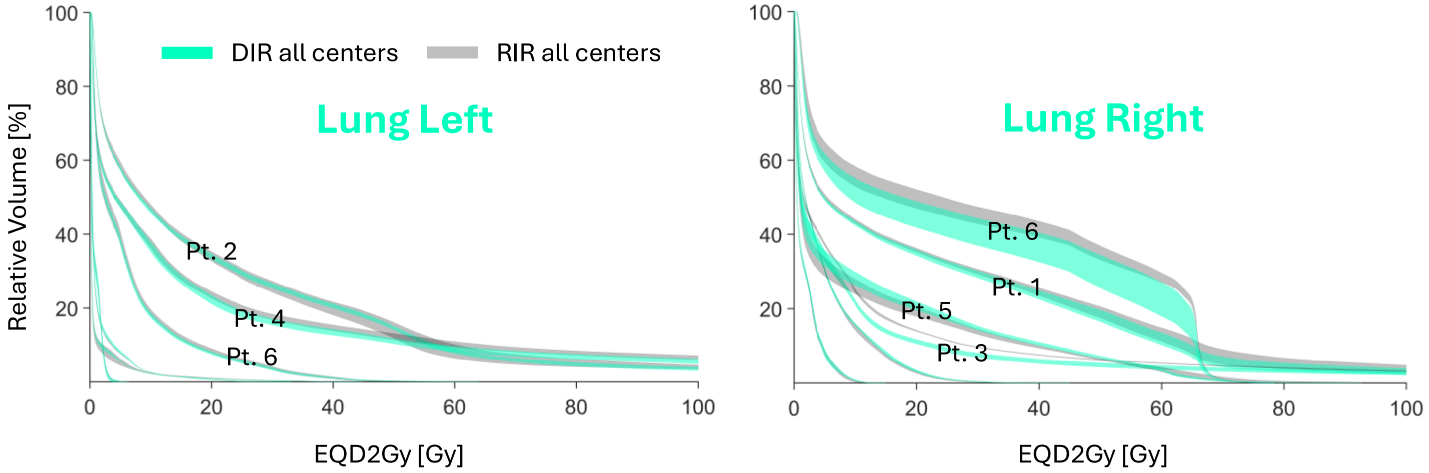

Supplementary Fig. S9. DVH bands for six OARs for cases (pt) 1-6, illustrating the minimum to maximum values across all centres for both RIR (grey) and DIR (coloured) for the cumulated EQD2Gy doses $D_{cum,current,EQD2}$ at $CT_{current}$.

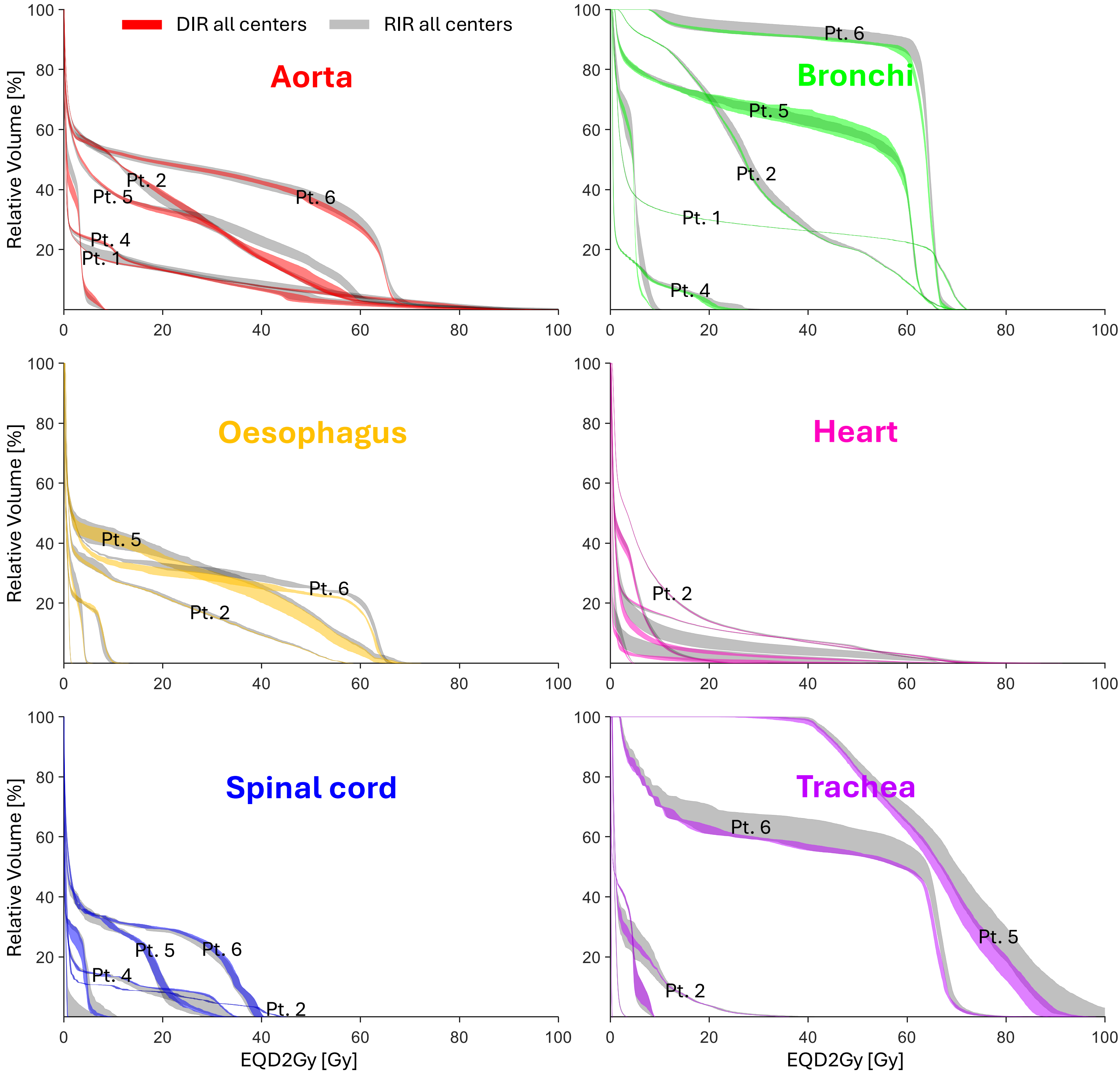

Supplementary Fig. S10. Unity plots of the paired RIR and DIR values for individual patients and OAR corresponding to the data (resolution: 1 Gy) in Fig. 3.

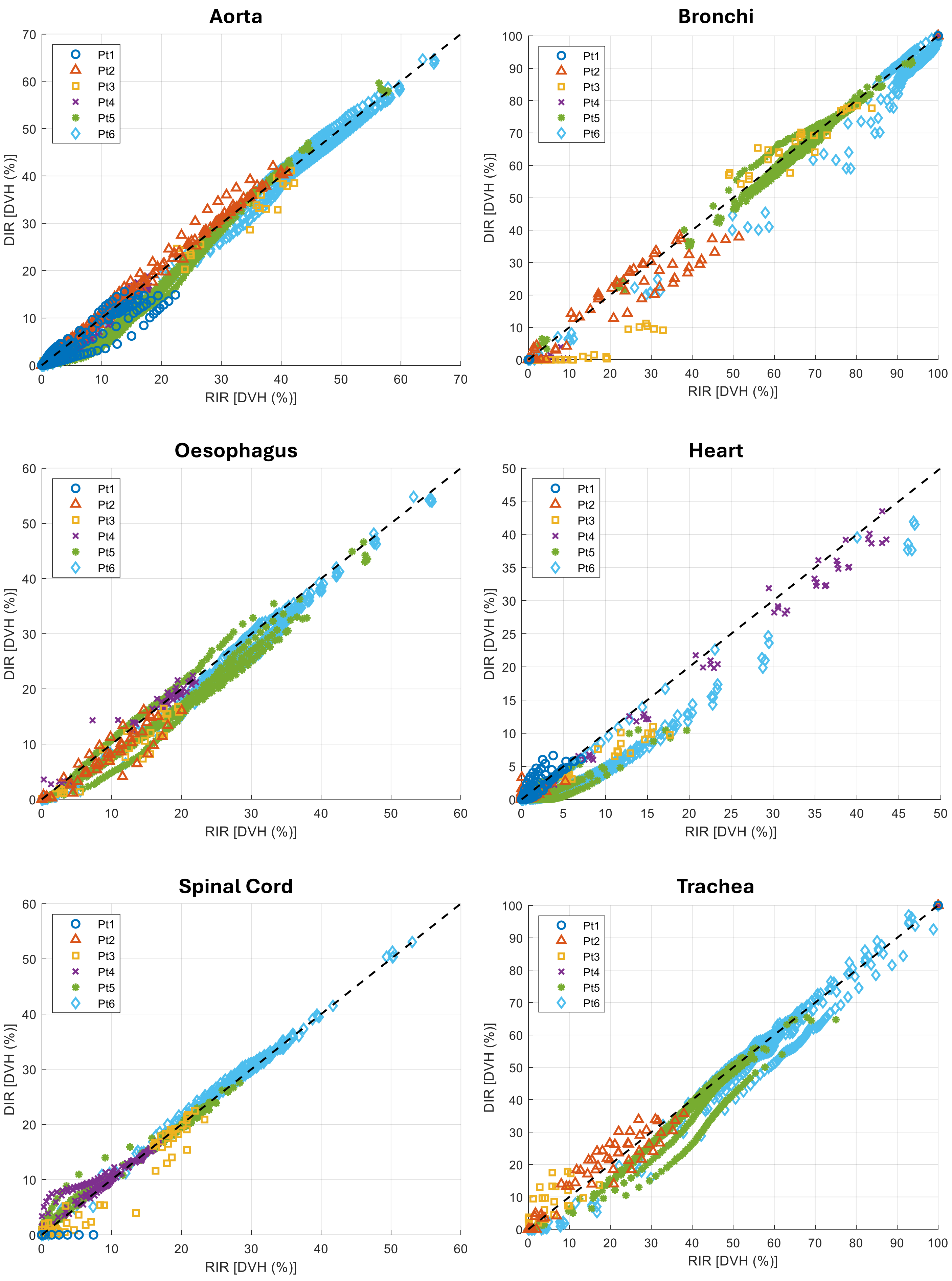

Supplementary Table S3. Areas within the interquartile range (IQR) of the full DVH bands for all patients and OARs for RIR and DIR, for both $D_{prev,EQD2}$ and $D_{cum,curr,EQD2}$.

$D_{prev,EQD2}$

**Patient**	1		2		3		4		5		6		
**Method**	DIR	RIR	DIR	RIR	DIR	RIR	DIR	RIR	DIR	RIR	DIR	RIR	**p-value**
**Aorta**	0,1126	0,8002	0,2826	0,4638	0,0424	0,0495	0,3426	0,3481	0,5685	0,7965	0,3741	0,8309	*0,0623*
**Bronchi**	0,0068	0,0648	0,1066	0,7853	0,0501	0,2638	0,0098	0,1705	0,5665	0,8067	0,3696	0,5680	0,0325
**Oesophagus**	0,0029	0,0188	0,0868	0,1062	0,0407	0,0956	0,0556	0,0559	0,8928	0,6164	0,4058	0,2558	*0,3377*
**Heart**	0,0363	0,2045	0,0255	0,1047	0,0156	0,0229	0,0623	0,0961	0,1470	0,8251	0,3045	0,2169	*0,2468*
**Spinal cord**	0,0047	0,0069	0,0131	0,0058	0,0542	0,1218	0,1012	0,3909	0,1544	0,1468	0,1857	0,2494	*0,2031*
**Trachea**	0,0054	0,0251	0,0463	0,4865	0,1461	0,0229	0,0006	0,0025	0,6809	2,1272	0,5996	0,7612	*0,2306*
**LungLeft**	0,0184	0,1022	1,3114	0,4717	0,0177	0,0054	1,8740	1,0717	0,0205	0,0113	0,2443	0,2296	*0,1921*
**LungRight**	1,4150	2,6831	0,0499	0,1117	0,5768	0,0887	0,0178	0,0242	0,6451	0,4061	2,3752	0,5961	*0,6480*

$D_{cum,curr,EQD2}$

**Patient**	1		2		3		4		5		6		
**Method**	DIR	RIR	DIR	RIR	DIR	RIR	DIR	RIR	DIR	RIR	DIR	RIR	**p-value**
**Aorta**	0,1234	0,7793	0,3071	0,9576	0,0678	0,0843	0,4438	0,5778	0,5599	0,8003	0,3783	0,8260	0,0227
**Bronchi**	0,0236	0,0654	0,1209	0,7621	0,0531	0,2621	0,0888	0,2345	0,5811	0,8394	0,3727	0,5670	0,0317
**Oesophagus**	0,0035	0,0180	0,0684	0,2162	0,0447	0,0950	0,0576	0,0812	1,0245	0,7626	0,4060	0,2518	*0,6445*
**Heart**	0,0697	0,2062	0,0323	0,1097	0,0152	0,0216	0,0757	0,1267	0,1479	0,8260	0,3048	0,2160	*0,2539*
**Spinal cord**	0,0333	0,0233	0,1066	0,1024	0,0556	0,1272	0,1063	0,4046	0,1918	0,3077	0,1918	0,2594	*0,1088*
**Trachea**	0,0051	0,0258	0,0555	0,4804	0,1445	0,0265	0,0068	0,0066	0,6750	1,9988	0,5905	0,7568	*0,2232*
**LungLeft**	0,0210	0,1045	1,3391	0,4899	0,0185	0,0101	1,9311	1,1967	0,0220	0,0161	0,2013	0,2568	*0,2228*
**LungRight**	1,4446	2,6363	0,0576	0,1404	0,5600	0,1565	0,0284	0,0472	0,6160	0,6406	2,3753	0,5998	*0,7296*

All areas are derived from the data shown in Figs. 4 and 5. P-values indicate statistical comparisons of DVH band areas between mapping methods. P-values in italic are not significant.
